# Endoscopically injectable and self‐crosslinkable hydrogel‐mediated stem cell transplantation for alleviating esophageal stricture after endoscopic submucosal dissection

**DOI:** 10.1002/btm2.10521

**Published:** 2023-04-18

**Authors:** Hyunsoo Chung, Soohwan An, Seung Yeop Han, Jihoon Jeon, Seung‐Woo Cho, Yong Chan Lee

**Affiliations:** ^1^ Department of Internal Medicine and Liver Research Institute Seoul National University College of Medicine Seoul Republic of Korea; ^2^ Department of Medical Device Development Seoul National University College of Medicine Seoul Republic of Korea; ^3^ Yonsei University Graduate School of Medicine Seoul Republic of Korea; ^4^ Department of Biotechnology Yonsei University Seoul Republic of Korea; ^5^ Center for Nanomedicine, Institute for Basic Science (IBS) Seoul Republic of Korea; ^6^ Graduate Program of Nano Biomedical Engineering (NanoBME) Advanced Science Institute, Yonsei University Seoul Republic of Korea; ^7^ Department of Internal Medicine Yonsei University College of Medicine Seoul Republic of Korea

**Keywords:** adipose tissue‐derived stem cell, endoscopic injection, endoscopic submucosal dissection, esophageal stricture, in situ forming hydrogel, self‐crosslinkable hydrogel

## Abstract

Esophageal stricture after extensive endoscopic submucosal dissection impairs the quality of life of patients with superficial esophageal carcinoma. Beyond the limitations of conventional treatments including endoscopic balloon dilatation and the application of oral/topical corticosteroids, several cell therapies have been recently attempted. However, such methods are still limited in clinical situations and existing setups, and the efficacies are less in some cases since the transplanted cells hardly remain at the resection site for a long time due to swallowing and peristalsis of the esophagus. Thus, a cell transplantation platform directly applicable with clinically established equipment and enabling stable retention of transplanted cells can be a promising therapeutic option for better clinical outcomes. Inspired by ascidians that rapidly self‐regenerate, this study demonstrates endoscopically injectable and self‐crosslinkable hyaluronate that allows both endoscopic injection in a liquid state and self‐crosslinking as an in situ‐forming scaffold for stem cell therapy. The pre‐gel solution may compatibly be applied with endoscopic tubes and needles of small diameters, based on the improved injectability compared to the previously reported endoscopically injectable hydrogel system. The hydrogel can be formed via self‐crosslinking under in vivo oxidative environment, while also exhibiting superior biocompatibility. Finally, the mixture containing adipose‐derived stem cells and the hydrogel can significantly alleviate esophageal stricture after endoscopic submucosal dissection (75% of circumference, 5 cm in length) in a porcine model through paracrine effects of the stem cell in the hydrogel, which modulate regenerative processes. The stricture rates on Day 21 were 79.5% ± 2.0%, 62.8% ± 1.7%, and 37.9% ± 2.9% in the control, stem cell only, and stem cell‐hydrogel groups, respectively (*p* < 0.05). Therefore, this endoscopically injectable hydrogel‐based therapeutic cell delivery system can serve as a promising platform for cell therapies in various clinically relevant situations.

## 1 INTRODUCTION

Endoscopic submucosal dissection (ESD) is generally accepted as a minimally invasive treatment for superficial esophageal cancer.^1^, ^2^ However, esophageal strictures that occur after extensive ESD may impair with the quality of life of patients. If more than 75% of the esophageal circumference of the mucosa is resected, the risk of esophageal stricture increases to 66%–88%. In particular, in patients with mucosal defects in 100% of the luminal circumference, an average of 33.5 sessions of endoscopic balloon dilatation procedures are required to treat esophageal stricture.^3^ To prevent stenosis after ESD, conventional treatment includes endoscopic balloon dilatation and the application of oral/topical corticosteroids.^4^ Although these methods are effective, adverse events such as perforation, mediastinal abscess, and steroid‐induced side effects are concerning. Recently, cell sheet transplantation has been attempted; however, despite its favorable effects, this method still has limited clinical applications.^5^ Efforts to improve stenosis using adipose‐derived stem cells (ADSCs) are also underway, and favorable results have been reported in animal experiments.^6^, ^7^, ^8^ However, the potential for early loss of ADSCs due to swallowing and peristalsis has been revealed as a shortcoming of ADSC‐mediated procedures. Therefore, better results are expected if ADSCs can remain at the resection site for a longer period. We previously developed and reported a bio‐inspired self‐crosslinkable hydrogel system with excellent biocompatibility and tissue adhesiveness.^9^ This system can be applied to medical fillers for volumetric augmentation and wrinkle correction by the superior injectability and in vivo mechanical stability.^10^ The injectability should be more critical for endoscopy which consists of long and narrow tubing with very small gauge needle.^11^, ^12^ Thus, we predicted that our injectable hydrogel system could be effectively introduced in the clinical setting via endoscopy equipment and that incorporating ADSCs into the hydrogel system might enhance the retention and therapeutic effects of ADSCs. This study aimed to investigate whether endoscopic injection of an ADSC‐hydrogel mixture can prevent post‐ESD esophageal strictures.

## 2 MATERIALS AND METHODS

### 2.1 Synthesis of endoscopically injectable and self‐crosslinkable hyaluronate

Endoscopically injectable and self‐crosslinkable hyaluronate (EISCH) was synthesized by conjugating pyrogallol (PG) group to hyaluronate via carbodiimide chemistry, according to previously reported procedures, with slight modifications.^10^ Sodium hyaluronate (HA; Molecular weight 200 kDa, Lifecore Biomedical, IL, USA) was dissolved in triple‐distilled water (TDW) at a concentration of 10 mg/mL. 1‐(3‐Dimethylaminopropyl)‐3‐ethylcarbodiimide hydrochloride (EDC; Thermo Fisher Scientific, Waltham, MA, USA) was added to the HA solution at 1.5 molar ratio to HA and stirred for 15 min. Then, N‐hydroxysuccinimide (NHS; Sigma‐Aldrich, St. Louis, MO, USA) was added to the HA/EDC solution at an equal molar ratio to HA. After reacting for 15 min, 5‐hydroxydopamine was added a little at a time into the solution at an equal molar ratio to HA and reacted for 24 h at room temperature, while maintaining the pH level around 4.5 using 0.5 M NaOH solution. Then, unreacted reactants and byproducts were removed by dialysis using a Cellu Sep T2 dialysis membrane with a MW cut‐off of 6–8 kDa (Membrane Filtration Products Inc., Seguin, TX, USA) in acidic phosphate‐buffered saline (PBS; pH 4.3, Biosesang, Seongnam, Korea) and TDW. The synthesized EISCH was then lyophilized and stored at 4°C until use. The successful conjugation of 5‐hydroxydopamine to HA was confirmed using ^1^H‐NMR (300 MHz, Bruker, Billerica, MA, USA) and ultraviolet‐visible (UV‐vis) light spectrophotometer (JASCO Corporation, Tokyo, Japan). Degree of substitution (DS) of EISCH was measured using ^1^H‐NMR by calculating the integral area ratio of the peak of aromatic protons of PG groups in the conjugated 5‐hydroxydopamine to those of the methyl groups of the HA backbone.

### 2.2 Preparation and material characterization of EISCH hydrogel

To induce in vivo‐mimetic crosslinking of the EISCH, the lyophilized conjugate was dissolved in neutral PBS (pH 7, Sigma‐Aldrich) and evenly mixed with different concentrations of horseradish peroxidase (HRP; Sigma‐Aldrich) solution. The final concentration of EISCH adjusted to 2 w/v%, and that of HRP was set to predetermined concentrations (0.06, 0.6, and 6 U/mL, respectively). For in vivo crosslinking, the pre‐gel solution was prepared by dissolving lyophilized conjugate in PBS at a concentration of 2 w/v% and injected directly into desired tissues in vivo. The formed hydrogel via in vivo crosslinking was retrieved for observation and further analysis. The rheological analyses were performed using a rheometer (MCR 102, Anton Paar, Ashland, VA, USA). Viscoelastic properties of the EISCH hydrogels were examined by measuring storage (G′) and loss modulus (G″) in a frequency sweep mode at a frequency range of 0.1 to 1 Hz. The elastic modulus of the hydrogel was determined by calculating the average of storage moduli of each hydrogel at 1 Hz (*n* = 3–4). The elasticity (tan δ) of the hydrogels was determined by calculating the ratio of the loss modulus to storage modulus (G″/G′) at 1 Hz (*n* = 3–4). The images of scanning electron microscope (SEM) were obtained using a field emission scanning electron microscope (FE‐SEM; JEOL‐7001F, JEOL Ltd., Tokyo, Japan). The samples for SEM were prepared by lyophilizing the hydrogels formed by each condition and coating them with platinum (coating regime: 20 mA current, 1 min) using a Cressington sputter coater 208HR (Cressington Scientific Instruments, Watford, UK).

### 2.3 Injectability test of EISCH

The injectability of EISCH was evaluated by comparing the force needed for injecting EISCH with that of the previously reported endoscopically injectable shear‐thinning hydrogel based on alginate‐Laponite (Alg‐LP).^13^ The Alg‐LP hydrogel was prepared by mixing 0.2% of sodium alginate (Pronatal LF10/60, FMC Biopolymer, Philadelphia, PA, USA) solution with 2 mg/mL of Laponite (LAPONITE® XLG, BYK‐Chemie GmbH, Wesel, Germany). The viscoelastic properties of Alg‐LP hydrogel were measured in the same way mentioned above. The EISCH conjugate dissolved in PBS at a concentration of 2 w/v% and the Alg‐LP hydrogel were individually loaded into a 1 mL syringe equipped with different needle gauges (23, 25, 27, or 29G). The extrusion force was measured by universal testing machine with ILC‐50 N load cell (UTM, Mecmesin, MultiTest 2.5‐i, Slinfold, West Sussex, UK) (*n* = 3–8). The plunger of syringe loaded with each material was compressed by a probe equipped on the UTM with a jig for syringe fixation, and the probe was moved at a rate of 3.5 mm/s. The break loose force and dynamic glide force was defined as the peak force required to start the movement of plunger and the average force required to maintain the movement of plunger after the initiation, respectively. The injection force was evaluated using the samples from different batches of material synthesis or preparation. The injectability and self‐crosslink‐ability after injection were also checked by loading and injecting the pre‐gel solution (2 w/v% of EISCH solution) using a 23‐gauge endoscopic injection needle (NM‐610 U‐0423, Olympus, Tokyo, Japan).

### 2.4 In vitro examination of biocompatibility of EISCH hydrogel and paracrine effects

Human ADSCs (StemPro® Human Adipose‐Derived Stem Cells, Invitrogen, Carlsbad, CA, USA) were cultured using MesenPRO‐RS™ medium (Invitrogen). To evaluate the biocompatibility of EISCH hydrogel in vitro, ADSCs were encapsulated in the hydrogels (1.0 × 10^6^ cells per 100 μL of hydrogel) and stained using Live/Dead viability/cytotoxicity kit (Invitrogen) following the manufacturer's protocol at 1, 3, 7, and 14 days of cell culture. Then, the stained ADSCs were observed using a fluorescence microscope (IX73, Olympus), and the ratio of viable cells (green) to dead cells (red) was quantified by manually counting the number of cells in each condition from the acquired images (*n* = 10). In Live/Dead analysis, the images for quantification were acquired from different samples through separate cell experiments. The cell viability was also examined after injecting the cell‐loaded pre‐gel solution using a 1 mL syringe equipped with different gauge of needles (23, 25, 27, and 29G). For investigation on paracrine effects of stem cells encapsulated in EISCH, ADSCs (8.0 × 10^5^ cells per 80 μL of hydrogel) were encapsulated in the hydrogels formed via two different in vivo‐mimetic crosslinking using 0.6 and 6 U/mL of HRP. Each hydrogel containing ADSCs was incubated with MesenPRO‐RS™ medium, and the medium used for culture was collected at pre‐determined time points (2, 4, 7, 10, 14 days after encapsulation). The retrieved medium was used for quantifying the factors released from the encapsulated cells (*n* = 3–4), and the concentration of each factor (Vascular endothelial growth factor [VEGF] and Interleukin 10 [IL‐10]) was measured using a human VEGF ELISA kit and human IL‐10 ELISA kit (R&D Systems, Minneapolis, MN, USA) following the manufacturer's protocol. Independent material and cell samples were used for the ELISA analysis.

### 2.5 Confirmation of in vivo self‐crosslink‐ability of EISCH

The experimental protocol for the mouse experiments was approved by the Institutional Animal Care and Use Committee (IACUC) of Yonsei University (approval number: IACUC‐A‐201908‐946‐01). To confirm in vivo self‐crosslink‐ability of EISCH, the pre‐gel solution prepared by dissolving the conjugate at a concentration of 2 w/v% was loaded into a syringe with the needle narrower than clinically used endoscopic needle (26‐gauge needle), and then injected into subcutaneous tissues of mouse. The injected region was visually observed both immediately and 1 day after injection. Then, the mouse was sacrificed, and the injected region was surgically opened for observing the in vivo‐crosslinked EISCH hydrogel. Subsequently, the hydrogel with adjacent tissues was removed together and used for further rheological and histological analyses.

### 2.6 Cell transplantation test in a mouse model and histological analyses

To preliminarily test cell transplantation, ADSCs were labeled using Qtracker 655 cell labeling kit (Invitrogen) following the manufacturer's protocol. The labeled ADSCs (1.0 × 10^6^ cells per 100 μL of hydrogel) were resuspended in the pre‐gel solution (2 w/v% of EISCH solution) and injected into subcutaneous tissues in mice using a syringe with 26‐gauge needle. The formed hydrogel with cells and adjacent tissues was retrieved together at predetermined time points (Days 7 and 14) and fixed with 10% (v/v) formalin (Sigma‐Aldrich). Red signal from the fluorescent Qdot inside the cells was observed using a confocal microscope (LSM 880, Carl Zeiss, Jena, Germany). The region of interest for observing the transplanted cells was determined to check only the location within the transplanted hydrogel. The adjacent tissues were excluded for observation to avoid background signals from tissue autofluorescence for precise quantification of cell retention. The number of red signals from Qdot per unit area was quantified by image‐based analysis using independent samples of hydrogel with tissue (*n* = 8).

After 1, 3, and 7 days of injection, the mice were sacrificed, and the tissues with crosslinked hydrogels were retrieved for histological analysis. The tissues were fixed with 10% (v/v) formalin and processed with serially diluted sucrose solutions (15 and 30%) for cryoprotection (Sigma‐Aldrich). The prepared tissue‐embedded OCT blocks were sectioned at 6‐μm thickness and stained with hematoxylin (Sigma‐Aldrich) and eosin Y (Samchun Chemicals, Seoul, Korea) (H&E) for confirming successful in vivo crosslinking and Toluidine Blue O (Sigma‐Aldrich) for evaluating biocompatibility in vivo.

### 2.7 ESD procedure in a porcine model

The experimental protocol for the porcine experiment was approved by the IACUC of Yonsei University Health System (approval number:2019‐0129) and conducted in a dedicated animal facility. Fifteen female domestic pigs weighing 20–25 kg were used. Traditionally, 20–25 kg female pigs have been preferred for endoscopic and surgical animal experiments using pigs. This is because the growth rate of female pigs is slower than that of male pigs, allowing for observation for a long period of time. In particular, the size of the esophagus and stomach of 20–25 kg pigs is most similar to that of humans. They were fasted for 24 h before the procedure; however, water intake was allowed ad libitum. This study followed the ARRIVE reporting guidelines. Pigs were housed singly in cages and cared for according to the Guide for the Care and Use of Laboratory Animals. In detail, a sufficient space to turn or move freely was provided, and suspended flooring was used to prevent contamination by urine and feces.^14^ Pigs were randomly assigned to different three groups (control group, ADSC group, and ADSC‐EISCH group, *n* = 5). To minimize the unnecessary use of animals, five pigs were used in each group (2 on 7 days after ESD for early response evaluation, 1 on 14 days after ESD for intermediate evaluation, and 2 on 21 days after ESD for late response evaluation).

Atropine sulfate (0.05 mg/kg) was injected to the pigs intramuscularly. They were then anesthetized with 15 mg/kg ketamine hydrochloride and 3 mg/g xylazine hydrochloride, and then anesthetized with an intramuscular injection of 1–3 mg/kg alfaxalone. After endotracheal intubation, isoflurane and nitrous oxide gas were used to maintain anesthesia during the procedure under mechanical ventilation. A single‐channel esophagogastroduodenoscope (GIF‐Q260, Olympus) with a transparent attachment hood was fitted to the tip (D‐201‐11304, Olympus). Extensive esophageal ESD (75% of the circumference, 5 cm in length) was performed between 30 and 35 cm from the dental arch using a DualKnife (KD‐650Q, Olympus). En bloc resection was then performed. An electrosurgical generator (ForceTriad; Medtronic, Minneapolis, MN, USA) was set to the pure cut mode (30 W) or forced coagulation mode (35 W). All ESD procedures were performed by an experienced endoscopist (HC), who had performed more than 200 esophageal ESD procedures in humans.

### 2.8 Endoscopic injection of ADSCs and ADSC‐EISCH mixture after ESD

Immediately after ESD, the labeled ADSCs (1.0 × 10^6^ cells per 100 μL) were resuspended in PBS (ADSC group) and the pre‐gel solution (2 w/v% of EISCH solution) (ADSC‐EISCH group) and injected into residual submucosal tissues using a syringe with a 23‐gauge needle. Between 0.2 and 0.5 mL of the ADSC‐loaded EISCH solution per dose (total volume, 2 mL) was injected evenly throughout the lesion. Injecting solutions into the remaining submucosa is difficult and dangerous because the submucosa is very thin. Therefore, prior to injection, careful examination of the ESD site is essential. The first injection was done at the point where the remaining submucosal layer was clearly visible. Once the first injection was done, the submucosal layer of the injection area got swollen so that subsequent injections could be done safely at the margins of the first injection. In this way, the injection site was gradually enlarged and was able to cover the ESD site. It is also important to approach the needle diagonally, not vertically, into the submucosa for effective and safe injection. For postoperative care, all pigs were fed a liquid diet 24 h after ESD and a semi‐solid diet the following day.

### 2.9 Evaluation of the degree of esophageal stricture after ESD

On Days 7, 14, and 21 after ESD, the degree of stricture was evaluated using endoscopic and fluoroscopic examinations. Fluoroscopic evaluation was performed by injecting water‐soluble contrast (Gastrografin, Bracco, Milan, Italy) media through endoscopic working channel using C‐arm (GE OEC 9800, GE Healthcare, Chicago, IL, USA). By observing the contrast passage through the esophagus, the inner diameter of the esophagus can be checked, and the presence, length, and severity of stricture can be evaluated. After evaluation, pigs were sacrificed via intravenous injection of KCl in each group for macroscopic, histological, and immunofluorescence evaluations. After scheduled sacrifice on Days 7, 14, and 21, the resected esophagi were immediately placed on a polystyrene foam board and fixed using pins. The degree of stricture at the lesion site was expressed as the lateral mucosal constriction rate, calculated using the following formula, as previously described^7^, ^15^, ^16^:

$$\text{Mucosal constriction rate}\left(\;,(\%)\right)=\left[1-(\text{length of the short axis at the site of maximal constriction}\right)/(\text{length of the short axis at the normal mucosal site}\;\text{on the upper side}\;+\;\text{length of the short axis}\;\;\mathrm{at}\;\text{the normal mucosal site}\;\;\text{on the lower side})/2]\times 100.$$

To prevent selection bias, the endoscopists, radiologists, and pathologists have been blinded to the group assignments to evaluate the level of strictures endoscopically and with fluorography.

### 2.10 Histological and immunofluorescence analysis of the porcine model

Esophageal tissues were fixed in a 10% formaldehyde/saline solution, embedded in paraffin, and cut into 5‐μm sections. Tissue sections were stained with hematoxylin and eosin (H&E) and Masson's trichrome (MT) to examine histopathological changes, including fibrosis. Fluorescence microscopy was performed to track the ADSCs in the resected esophagi. Tissue sections were stained with anti‐α‐smooth muscle actin (α‐SMA) antibody (mouse, monoclonal antibody, Abcam, Cambridge, UK) for 60 min, anti‐myeloperoxidase (MPO) antibody (rabbit, polyclonal antibody, Abcam, Cambridge, UK) for 40 min, anti‐Ki‐67 antibody (rabbit, monoclonal antibody, Invitrogen, Carlsbad, CA, USA) for 60 min at room temperature, and anti‐VEGF antibody (rabbit, polyclonal antibody, Abcam, Cambridge, UK) overnight at 4°C. The samples labeled with the primary antibodies were incubated with an Alexa Fluor 488 goat anti‐mouse IgG (Thermo Fisher Scientific) or Alexa Fluor 594 goat anti‐rabbit IgG (Thermo Fisher Scientific), and then visualized with VECTASTAIN® Elite® ABC HRP system (Vector Laboratories, Burlingame, CA, USA) and DAB HRP substrate (Vector Laboratories) according to the manufacturer's protocols. The stained tissue samples were observed with a laser scanning confocal microscope (LSM 880, Carl Zeiss) or slide scanner (VS120‐S5‐W, Olympus). For image‐based quantification, each image was acquired from different tissue samples.

### 2.11 Statistical analysis

All quantitative data were expressed as the mean ± standard deviation (SD). Statistical analysis was performed to determine statistical significance by calculating the *p*‐values using Student's *t*‐test, Mann–Whitney *U* test for non‐parametric values, and analysis of variance (ANOVA) (GraphPad Software, CA, La Jolla, USA). *p* Values <0.05, 0.01, or 0.001 were considered statistically significant.

## 3 RESULTS

### 3.1 Material design and characteristics of EISCH for facile and effective cell delivery

**FIGURE 1:**
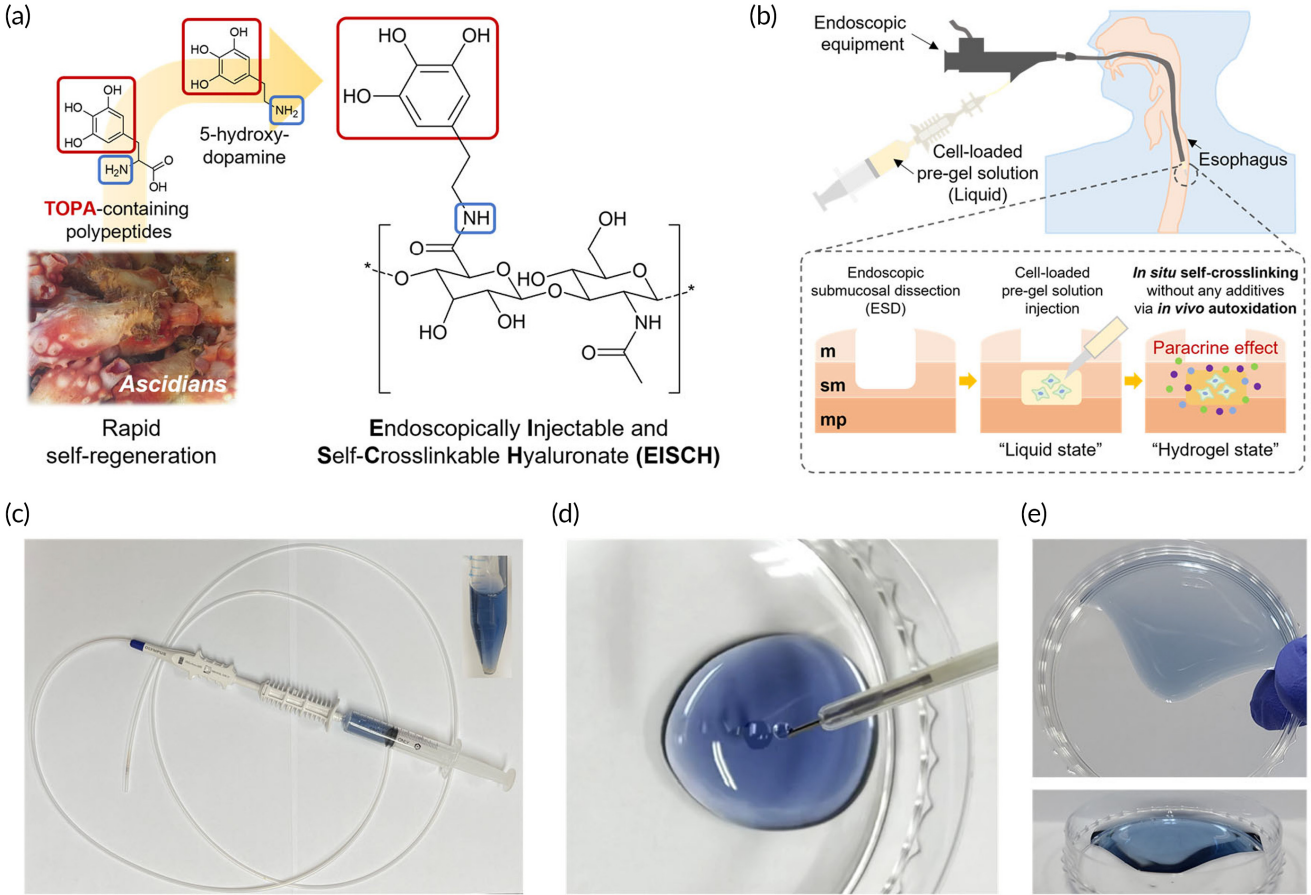
Material for endoscopic stem cell delivery. (a) Material design strategy and chemical structure of endoscopically injectable and self‐crosslinkable hyaluronate (EISCH). (b) Schematic illustration of the endoscopic injection of stem cell‐loaded EISCH into the esophageal submucosa after endoscopic submucosal dissection. m, mucosa; mp, Muscularis propria; sm, submucosa. Photographs of (c) an EISCH‐loaded syringe equipped with an endoscopy apparatus, (d) EISCH released from the needle through endoscopy tubing, and (e) EISCH hydrogel crosslinked via autooxidation after release.

The EISCH was designed from a highly autoxidative chemical moiety (PG, red) inspired by 3,4,5‐Trihydroxyphenylalanine (TOPA)‐containing polypeptides in a marine ascidian capable of rapid self‐regeneration (Figure 1a).^9^ HA, one of the most widely used extracellular matrix components in biomedical application, was utilized as a polymer backbone of this hydrogel system due to its great biocompatibility and biodegradability.^17^, ^18^ As HA is known to perform various important biological functions during tissue regeneration, it has been used to develop injectable hydrogel systems for wound healing.^19^, ^20^ The bioinspired polymer was synthesized by chemically conjugating 5‐hydroxydopamine containing PG group to the HA backbone via a carbodiimide coupling reaction. The conjugation of 5‐hydroxydopamine to carboxyl group in HA backbone was confirmed by ^1^H nuclear magnetic resonance (NMR) spectroscopy. The presence of proton peaks at ~6.4 ppm indicating aromatic protons in the PG groups demonstrated successful synthesis of EISCH conjugate (Figure S1a). The DS of EISCH was measured using ^1^H‐NMR by calculating the integral area ratio of the peak of aromatic protons in the PG groups to that of the methyl groups of the HA backbone. The DS was determined to be approximately 8%. Furthermore, the absorbance spectrum and its peak at 278 nm using UV‐vis spectroscopy also showed the presence of PG group within the polymer (Figure S1b). Initially, the EISCH liquid may be prepared as a cell‐loaded pre‐gel solution which is more suitable for easily passing narrow tubing and needle for injection than already formed hydrogel scaffold (Figure 1b). Immediately after in vivo injection, EISCH undergoes spontaneous self‐crosslinking without any complex procedures like mixing additives for crosslinking owing to the auto‐oxidative property of the conjugated PG group, which leads to in situ hydrogel formation, enabling effective cell retention as a scaffold. We confirmed that the system is practically applicable to a clinically used endoscopic equipment (Figure 1c). The EISCH solution (colored blue for better visualization) loaded in a syringe with injector could be released passing the tubing and needle without difficulty (Figure 1d). Then, the released EISCH solution was spontaneously self‐crosslinked to form the stable hydrogel, not flowing down (Figure 1e). Altogether, EISCH is considered a powerful endoscopically injectable hydrogel platform for facile and effective cell transplantation, and thus EISCH was used for endoscopic transplantation of therapeutic stem cells to a defective esophagus in this study.

### 3.2 Injectability of EISCH system

**FIGURE 2:**
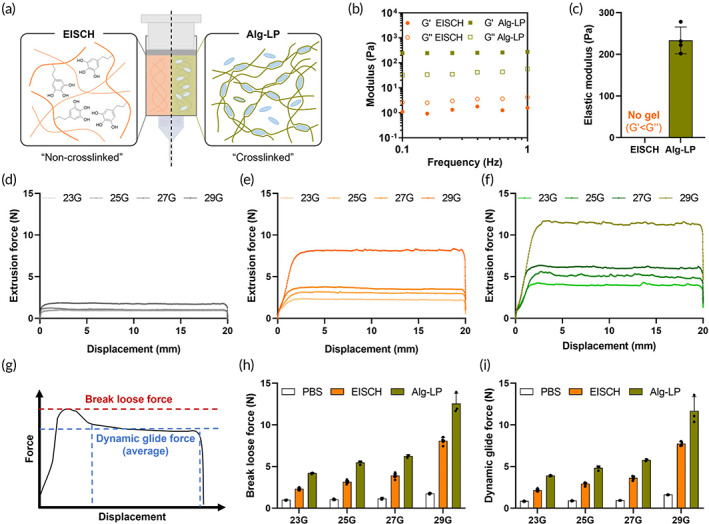
Improved injectability of endoscopically injectable and self‐crosslinkable hyaluronate (EISCH). (a) Schematic illustration of two types of endoscopically injectable hydrogels. (b) Rheological properties of both hydrogel systems before injection measured in a frequency sweep mode. G′: Storage modulus, G″: Loss modulus. (c) Average elastic moduli of both hydrogel systems before injection (*n* = 4, independent samples). Forces required to push the plunger of syringes containing (d) phosphate‐buffered saline (PBS), (e) EISCH, and (f) alginate‐Laponite (Alg‐LP), equipped with different gauges of needles (23, 25, 27, and 29G). (g) Schematic description for calculating break loose force and dynamic glide force from the measured force‐displacement graph. Average forces required (h) to initiate and (i) to sustain the movement of plunger of syringe containing PBS, EISCH, and Alg‐LP, equipped with each gauge of needles (23, 25, 27, and 29G) (*n* = 3–8, independent samples from different synthesis and preparation batches).

The injectability of hydrogels is critical in terms of practical and clinical availability.^21^ It would especially be a key factor when the hydrogel is applied as an injecting solution for an endoscopic device that consists of a tubing and needle with a very narrow gauge. The Alg‐LP hydrogel system, which is the previously reported hydrogel system with shear‐thinning‐based injectability for endoscopic injection, was demonstrated with the potential to serve as submucosal injection fluids for cushion development to facilitate polyp removal.^13^ The Alg‐LP system consists of alginate and smectite clays called Laponite, which is prepared as a crosslinked hydrogel form immediately after mixing the components, in contrast with the EISCH system prepared as a non‐crosslinked liquid state (Figure 2a). The rheological properties of the Alg‐LP showed its state as a viscoelastic solid (G′ > G″) having elastic modulus over 200 Pa, while the EISCH showed the rheological behavior as a liquid (G′ < G″) (Figure 2b,c). Thus, the injection force of the Alg‐LP to involve inducing shear‐thinning should be higher than those of EISCH; otherwise, larger size of needle should be needed for the injection of Alg‐LP. Clinically, high pressure during injection may cause severe complications including vascular/neural damage, and the large perforation resulted from using large needle increases the opportunity of bleeding, pain, and subsequent infection.^22^, ^23^, ^24^ Both are not only undesirable, but also especially unsuitable for endoscopic injection using small‐gauge needle. To comparatively evaluate the injectability, each hydrogel system was loaded in a syringe equipped with 23, 25, 27, and 29 G needles and pressed by the probe with UTM for measurement of the injection force. In the PBS and EISCH groups, the shape and roughness showed in the force‐displacement graphs were relatively smooth, which might be attributed to their rheological properties of liquid state (Figure 2d,e). However, those showed in the graphs in the Alg‐LP group were relatively rough because the crosslinked Alg‐LP hydrogel should be broken via shear‐thinning for passing the syringe needle (Figure 2f). Furthermore, the break loose force and dynamic glide force were evaluated (Figure 2g). Although both break loose force and dynamic glide force in the EISCH and Alg‐LP groups were higher than those in the PBS group, those in the EISCH group were significantly lower than those in the Alg‐LP group (Figure 2h,i). Quantitatively, EISCH required up to ~45% less force for injection compared to Alg‐LP using a same size of needle. Altogether, the in situ self‐crosslinkable EISCH system exhibited superior injectability than the shear‐thinning Alg‐LP system, which demonstrates it as an excellent candidate for endoscopic injection leading up to surgeons' technical comfort in actual use, improved accuracy of treatment and thus, better clinical outcomes.

### 3.3 Material properties of EISCH hydrogel

**FIGURE 3:**
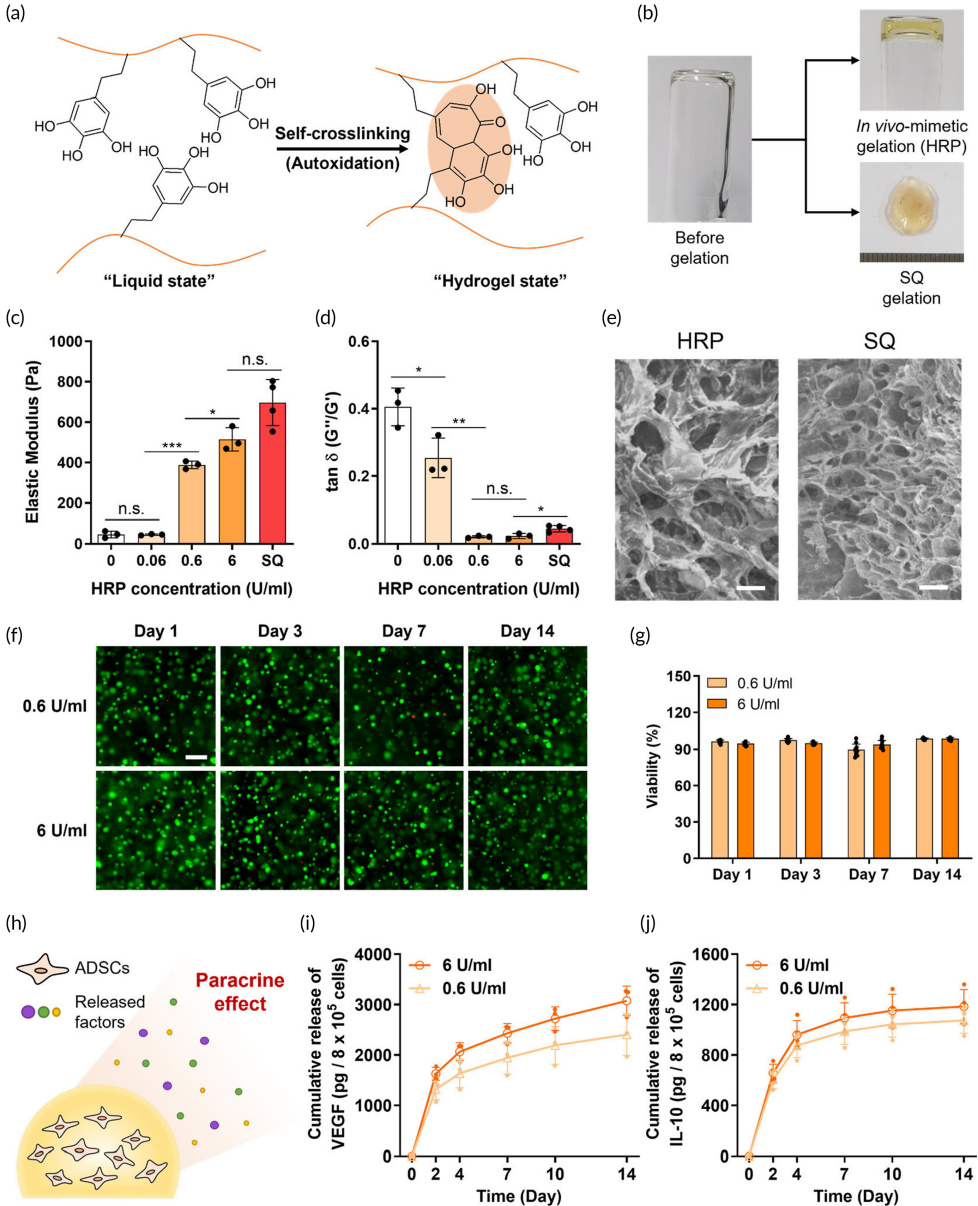
Self‐crosslink‐ability and in vitro cytocompatibility of endoscopically injectable and self‐crosslinkable hyaluronate (EISCH) hydrogel. (a) Crosslinking mechanism of the EISCH hydrogel via autoxidative coupling of its pyrogallol groups. (b) Photographs of the EISCH solution before gelation, EISCH hydrogel after in vivo‐mimetic gelation using horseradish peroxidase (HRP), and EISCH hydrogel retrieved after in vivo gelation in the subcutaneous tissue of a mouse (SQ gelation). (c) Average elastic moduli and (d) elasticity (tan *δ* [G″/G′]) of EISCH hydrogels spontaneously crosslinked without enzyme (0 U/mL group), crosslinked with different concentrations of HRP (0.06, 0.6, and 6 U/mL groups), and crosslinked in the subcutaneous tissue of a mouse (SQ group) (*n* = 3–4; *p < 0.05; **p < 0.01; ***p < 0.001, independent samples). (e) Internal structures of EISCH hydrogels crosslinked with HRP (6 U/mL, left) and crosslinked in in vivo condition (right). (f) Fluorescent images from the live/dead assay of adipose‐derived stem cells (ADSCs) encapsulated in the EISCH hydrogels crosslinked with different concentrations of HRP (0.6 and 6 U/mL) at predetermined timepoints (Days 1, 3, 7, and 14). (g) Viability of ADSCs encapsulated in the EISCH hydrogels quantified by calculating the number and ratio of live and dead cells in the images acquired in the live/dead assay (*n* = 10, independent samples through separate cell experiments). (h) Schematic illustration of the paracrine effects of the ADSCs encapsulated in the EISCH hydrogel. Release profiles of (i) vascular endothelial growth factor and (j) IL‐10 from the ADSCs encapsulated in the EISCH hydrogels crosslinked with different concentrations of HRP (0.6 and 6 U/mL) (*n* = 3–4, independent material and cell samples).

The EISCH hydrogel could be formed via self‐crosslinking due to autoxidative property of PG groups (Figure 3a).^10^ This oxidative crosslinking can be accelerated and enhanced by various oxidative molecules existing in in vivo oxidative environment.^25^ Thus, we used a peroxidase enzyme (HRP) for in vivo‐mimetic gelation since the enzyme was considered as one of the key factors for complex oxidative processes in vivo (Figure 3b).^26^, ^27^, ^28^, ^29^ The stability of the formed hydrogel was confirmed by a consistently higher storage modulus (G′) than loss modulus (G″) which were measured by the frequency sweep mode using a rheometer in the range from 0.1 to 1 Hz (Figure S2). For the analysis of the physical and rheological properties of the EISCH hydrogel, the storage and loss moduli at three different concentrations of HRP were measured using the rheometer. While the average storage modulus of EISCH hydrogel (at 1 Hz) formed with 0.06 U/mL of HRP was similar to that of the hydrogel without any enzyme, the modulus of those with 0.6 and 6 U/mL of HRP were significantly increased (Figure 3c). Of that, the hydrogel formed by using 6 U/mL of HRP even exhibited a similar level of physical property of the hydrogel made within an in vivo oxidative condition. Interestingly, several previous studies had shown that the concentration of HRP for enzymatic crosslinking mainly affects the gelation rate rather than the physical properties of the formed hydrogels, but the concentration of HRP comparatively influenced the physical properties of EISCH.^30^, ^31^ We assumed that peroxidase could be directly involved in and change the path of reaction of the galloyl groups in EISCH, and this phenomenon could also happen in an in vivo oxidative environment.^32^, ^33^ In contrast, hydrogels formed with different concentrations of HRP exhibited a reverse trend in the elasticity (tan δ, G″/G′), which decreased as the concentration of HRP was higher due to the mechanical reinforcement and stiffening by peroxidase (Figure 3d). Based on these results, EISCH crosslinked in the condition of 0.6 and 6 U/mL of HRP appeared to highly resemble the hydrogel formed within an in vivo condition in terms of physical and rheological properties. Furthermore, the internal structures of HRP‐crosslinked and in vivo‐crosslinked hydrogels seemed to be similar according to the images obtained from a SEM (Figure 3e). Thus, these crosslinking conditions to use 0.6 and 6 U/mL of HRP were chosen for the subsequent in vitro experiments.

### 3.4 In vitro cytocompatibility of EISCH hydrogel

First, we examined the cytotoxicity of the EISCH hydrogels in vitro, which is an important factor for stem cell transplantation and long‐term maintenance in vivo. Cellular viability was assessed through Live/Dead assay, and the ADSCs in the EISCH hydrogel crosslinked by in vivo‐mimetic crosslinking using HRP exhibited good viabilities at early time points (Days 1 and 3), which means the oxidative crosslinking of EISCH does not negatively affect the viability of the encapsulated cells (Figure 3f,g). Furthermore, the encapsulated ADSCs were highly viable even until Day 14 (about 90%), which demonstrates that stem cells could be effectively delivered to in vivo environments by utilizing the EISCH hydrogel system and successfully maintain their conditions for a long time within the hydrogel. Cell viability measured after passing the cell‐loaded EISCH pre‐gel solution through different gauge of needles (23, 25, 27, and 29G) was not significantly different (more than 90% viability in all groups) from control group (Figure S3a,b). Thus, the stress during cell injection using EISCH seems to have minimal effect on the viability of the ADSCs, which is likely attributed to the superior injectability of EISCH.

Next, we checked the paracrine effect of the stem cells encapsulated in the EISCH hydrogel, which was expected to contribute to therapeutic effects at transplanted regions (Figure 3h). ADSCs are well‐known for their ability to secrete various growth factors and cytokines for regeneration and immune modulation.^34^, ^35^, ^36^ We examined the paracrine effects of the 3D‐encapsulated ADSCs by measuring two well‐known proteins released from the ADSCs encapsulated in EISCH hydrogel which were formed by two conditions of in vivo‐mimetic crosslinking. VEGF, one of the representative growth factors important to regenerative process, was released from the ADSCs encapsulated in EISCH over 14 days after seeding although the amount gradually decreased (Figure 3i). Furthermore, interleukin 10 (IL‐10), one of the typical anti‐inflammatory cytokines, was also secreted from the ADSCs encapsulated in EISCH hydrogel, although the released amount of IL‐10 also decreased over time (Figure 3j). These results implies that the EISCH hydrogel could provide cyto‐compatible microenvironment for the encapsulated ADSCs enough to perform their own roles in paracrine effects.

### 3.5 In vivo crosslink‐ability and biocompatibility of EISCH hydrogel

**FIGURE 4:**
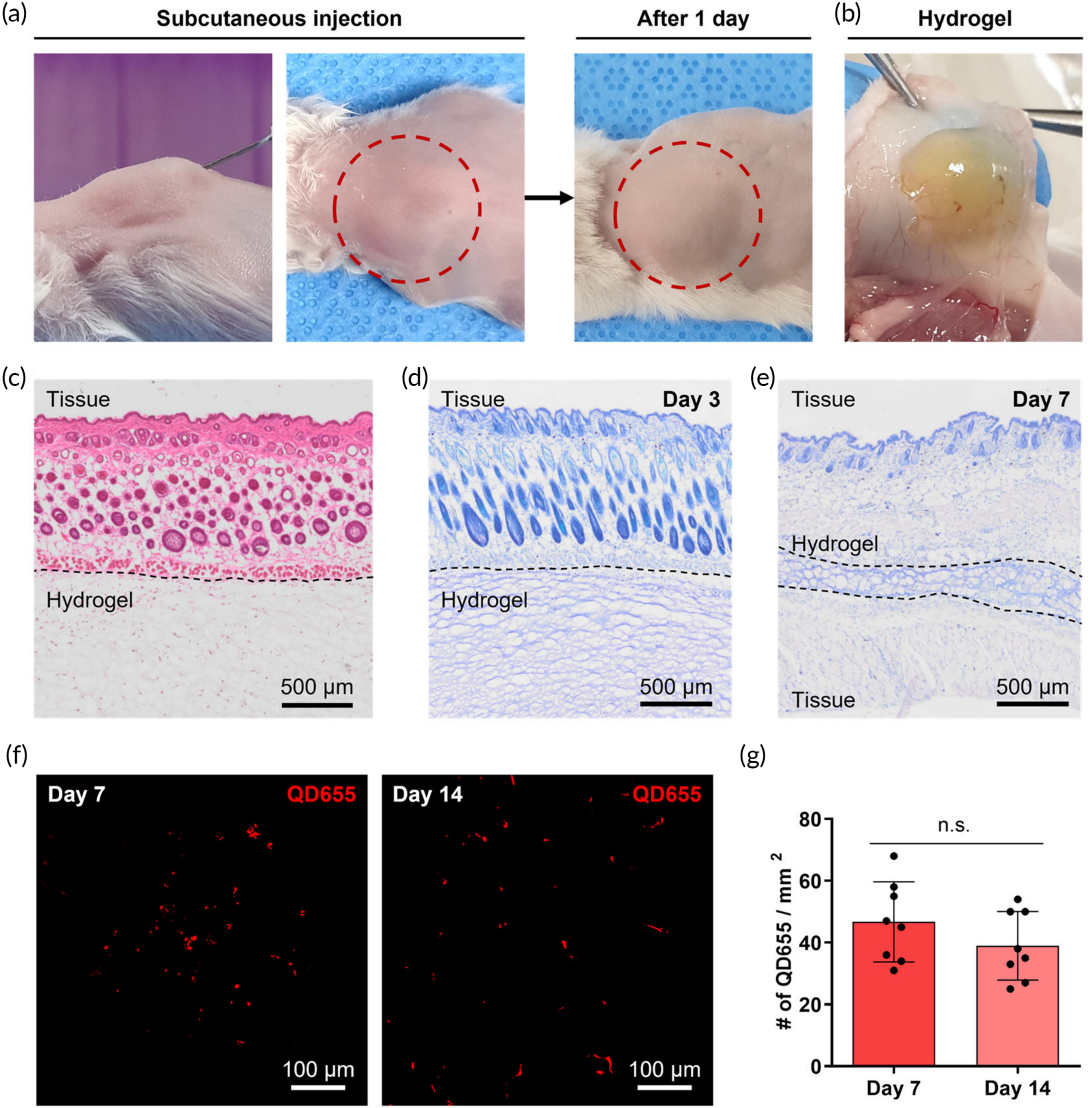
In vivo self‐crosslinking of endoscopically injectable and self‐crosslinkable hyaluronate (EISCH) and stem cell delivery using EISCH hydrogel in a mouse model. (a) Gross views of subcutaneously injected EISCH (left) and the injected region after 1 day (right). (b) In vivo self‐crosslinked EISCH hydrogel adhered onto the skin in a mouse. (c) H&E staining image at Day 1 and toluidine blue staining images at (d) Day 3 and (e) Day 7 of subcutaneous tissues after injection of EISCH. (f) Fluorescence images of QD655‐labeled adipose‐derived stem cells encapsulated in the EISCH and injected into the subcutaneous tissues in a mouse model after 7 days (left) and 14 days (right). (g) Quantification of the number of QD655‐positive cells per unit area at Days 7 and 14 (*n* = 8, independent samples of hydrogel with tissue).

For investigation of the injectability and in situ self‐crosslinking of EISCH, it was injected to subcutaneous tissue in mice. The pre‐gel solution was prepared by dissolving the lyophilized EISCH with PBS at a concentration of 2 w/v%. Then the liquid was subcutaneously injected using a 1 mL syringe with 26‐gauge needle. Although the solution was slightly viscous, it was easily released through the 26‐gauge needle which is narrower than clinically used endoscopic needles such as 22‐ to 25‐gauge needles. The injected solution did not instantly spread all over the surrounding subcutaneous regions due to its viscosity and maintained its own shape, making the injected region look swollen (Figure 4a). Then, the injected pre‐gel solution would be crosslinked by in vivo oxidative processes, maintaining its original shape and volume even 1 day after injection. Interestingly, the location of the formed hydrogel did not change at all, which might have resulted from the tissue‐adhesiveness of PG group and its intermediates during oxidative processes.^9^ In fact, the hydrogel was successfully crosslinked in the in vivo environment, and stably adhered to the surface of subcutaneous tissues which was confirmed when the injected region was surgically opened after 1‐day post‐injection (Figure 4b). Histological analysis also showed that the hydrogel was tightly integrated into the tissue, which was shown in the H&E staining image (Figure 4c). For assessing the in vivo biocompatibility of EISCH, the pre‐gel solutions were subcutaneously injected into mice, and then the crosslinked hydrogels and the adjacent tissue were removed altogether at the predetermined time points for histological analysis. Toluidine blue staining showed that the hydrogels did not induce problematic inflammatory responses in surrounding tissue at both early and late time points (Days 3 and 7) (Figure 4d,e).

### 3.6 In vivo stem cell delivery to subcutaneous tissue in mouse using EISCH hydrogel

Investigation of stem cell delivery capability using EISCH hydrogel was conducted before the experiments using a porcine model. To this end, the Qtracker 655 (red fluorescent Qdot 655; QD655)‐labeled ADSCs were loaded into EISCH pre‐gel solution and injected into subcutaneous tissues in mice. Subsequently, the hydrogels formed within the tissues were retrieved and fixed at 7 and 14‐day post‐injection. Then, the fluorescent Qdots inside the delivered cells were observed using a confocal microscope, and an image‐based quantification was performed using the acquired images. After 7 days of injection, a considerable amount of ADSC existed in the hydrogel, and the ADSCs still remained in the hydrogel even after 14 days of transplantation (Figure 4f). Although the number of cells per unit area slightly decreased, it was not statistically significant (Figure 4g).

### 3.7 Endoscopic, fluoroscopic, and macroscopic evaluation of esophageal stricture after ESD

**FIGURE 5:**
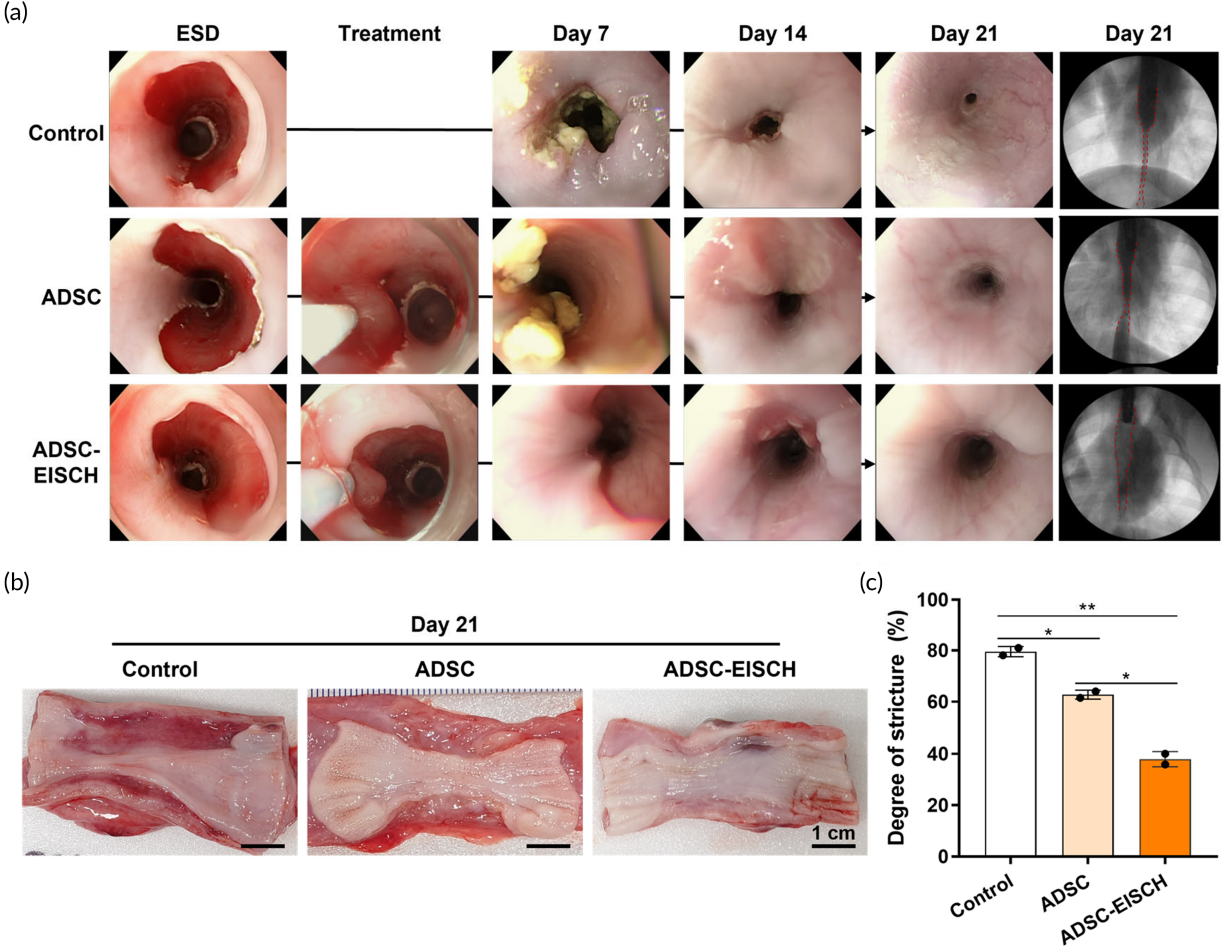
Evaluation of esophageal stricture level. (a) Serial endoscopic observation in all groups (Control, adipose‐derived stem cell [ADSC], ADSC‐endoscopically injectable and self‐crosslinkable hyaluronate [EISCH]) on Days 7, 14, and 21, and fluoroscopic analysis in all groups on Day 21 (b) Macroscopic observation of retrieved esophagus tissues on Day 21. Significant mucosal stricture was seen in the control group. The ADSC group showed milder stricture than the control group; however, it was more severe than the ADSC‐EISCH group. (c) Stricture rates determined by calculating the relative ratio of lengths of constricted and normal mucosal sites (*p < 0.05; **p < 0.01).

Esophageal ESD was performed safely in all pigs, but pig 14 (ADSC group) died unexpectedly on Day 6. Esophageal perforation with severe mediastinal inflammation was noted during the necropsy. Serial endoscopic findings were compared between the groups (Figure 5a). On Day 7, a large amount of exudate was observed to be attached to the ulcer base in the control group. However, a smaller amount of exudate with a cleaner ulcer base was observed in the ADSC and ADSC‐EISCH groups. On Day 14, severe esophageal strictures were observed in the control group, while the strictures appeared milder in the ADSC and ADSC‐EISCH groups; this pattern was more prominent on Day 21. Although the scope was not able to pass through the stricture in all groups, the degree of stenosis assessed by endoscopy and fluoroscopy was significantly milder in the ADSC and ADSC‐EISCH groups than in the control group. Macroscopic evaluation using resected esophagi on Day 21 revealed significant mucosal strictures in the control group (Figure 5b). The mean (±SD) stricture rates were 79.5% (±2.0%), 62.8% (±1.7%), and 37.9% (±2.9%) in the control, ADSC, and ADSC‐EISCH groups, respectively. Stricture rates were significantly milder in the ADSC and ADSC‐EISCH groups than in the control (*p* < 0.05 and *p* < 0.01, respectively) and milder in the ADSC‐EISCH group than in the ADSC group (*p* < 0.05) (Figure 5c).

### 3.8 Histological analysis of the esophagus after endoscopic injection of ADSC‐loaded EISCH hydrogels

**FIGURE 6:**
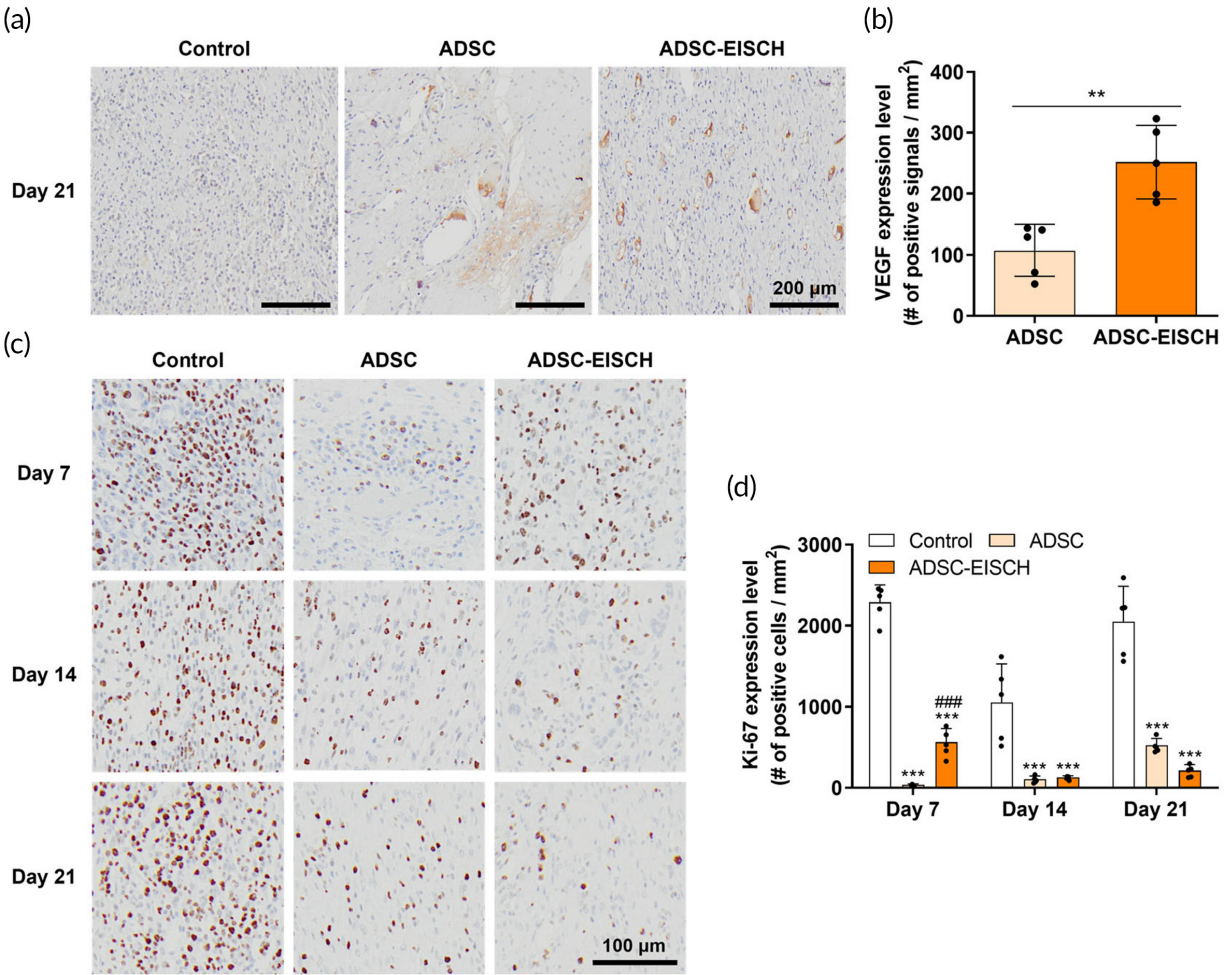
Immunohistochemical analysis of vascular endothelial growth factor (VEGF) and Ki‐67 expression. (a) Representative images of immunohistochemical staining (DAB) for VEGF on Day 21. (b) Quantification of DAB signals per unit area in the DAB staining images in the adipose‐derived stem cell (ADSC) and ADSC‐endoscopically injectable and self‐crosslinkable hyaluronate (EISCH) groups (*n* = 5; **p < 0.01). (c) Representative images of DAB staining for Ki‐67 and (d) image‐based quantification of the number of Ki‐67‐positive cells per unit area in the control group, ADSC group, and ADSC‐EISCH group after 7, 14, and 21 days post‐endoscopic submucosal dissection (*n* = 5; ***p < 0.001 vs. Control; ^###^ *p* < 0.001 vs. ADSC). For image‐based quantification, each image was acquired from different tissue samples.

**FIGURE 7:**
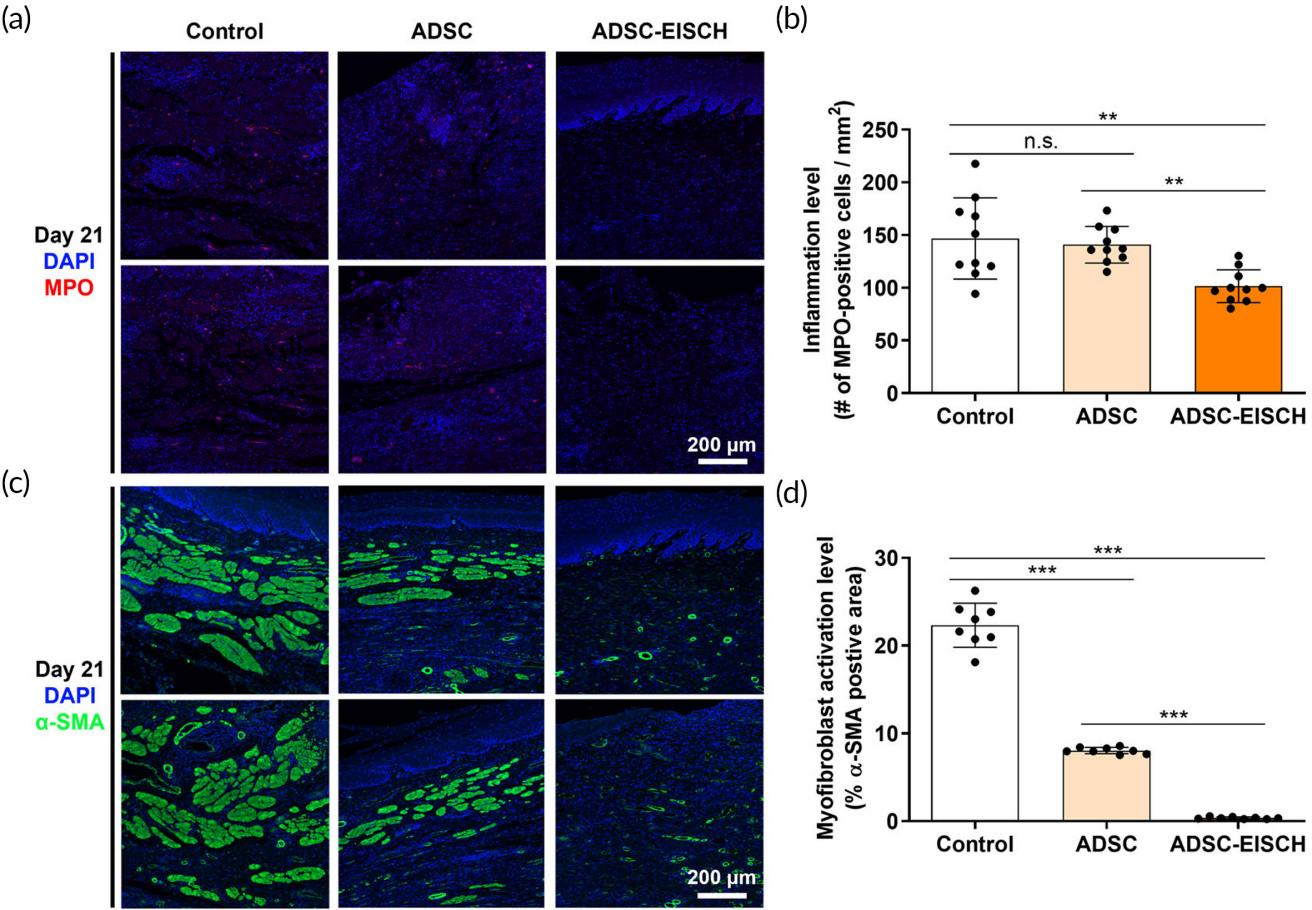
Immunohistochemical analysis for investigating the immune and fibrotic cell populations. (a) Representative images of immunofluorescence staining for myeloperoxidase (MPO) and (b) image‐based quantification of the number of MPO‐positive cells per unit area in the non‐treated (Control group), adipose‐derived stem cell (ADSC)‐treated (ADSC group), and ADSC‐loaded endoscopically injectable and self‐crosslinkable hyaluronate (EISCH)‐treated (ADSC‐EISCH group) esophagus after 21 days post‐endoscopic submucosal dissection (ESD) (*n* = 10; **p < 0.01). (c) Representative images of immunofluorescence staining for α‐SMA and (d) image‐based quantification of the percentage of MPO‐positive cells in the control, ADSC, and ADSC‐EISCH groups after 21 days post‐ESD (*n* = 8; ***p < 0.001). For image‐based quantification, each image was acquired from different tissue samples.

To trace the ADSC‐EISCH injection site, histological analysis of ADSC‐loaded EISCH‐injected esophageal tissues stained with H&E‐ and MT were performed using samples from pigs at 7 and 21 days after injection (Figure S4a,b). The injected gel was found to be attached to the esophageal tissue on Days 7 and 21. Confocal microscopy also revealed Q‐dots (QDs) in the gel, confirming the presence of ADSCs at the injection site (Figure S4c). The number of observed QDs within and between the ADSC group and ADSC‐EISCH group was compared over time (Figure S5). QD‐labeled cells were found in the submucosal layer in both the ADSC and ADSC‐EISCH groups until Day 21, although the number of QDs observed in both groups decreased over time. A higher number of QDs was observed in the ADSC‐EISCH group than in the ADSC group. To evaluate the effect of ADSC treatment on neovascularization helpful for tissue regeneration, we evaluated VEGF expression levels. ADSC‐EISCH group exhibited increased VEGF expression level compared to the control or ADSC groups on Day 21 (*p* < 0.05), which might also imply that the transplanted cells in ADSC‐EISCH group resided more in the submucosal layer at the time than those in ADSC group (Figure 6a,b). Moreover, the expression of Ki‐67, a cell proliferation marker, was significantly decreased in the ADSC and ADSC‐EISCH groups compared with the controls at each time point, suggesting that ADSCs inhibited the excessive proliferation in regeneration processes after ESD (Figure 6c). Although no statistical difference was observed between the ADSC and ADSC‐EISCH groups, the modulation effect seemed to be continued longer in ADSC‐EICSH group than ADSC group until 21 days (Figure 6d). In addition, MPO activity on Day 21 showed no difference between the control and ADSC groups, but it was decreased in the ADSC‐EISCH group compared with the control and ADSC groups (Figure 7a,b). Immunofluorescence staining for α‐SMA, a marker for myofibroblasts, showed significantly decreased expression of α‐SMA in the submucosal area in the ADSC and ADSC‐EISCH groups compared with the controls. The ADSC‐EISCH group showed a significant reduction compared with the ADSC group (Figure 7c,d).

## 4 DISCUSSION

As stricture formation after large‐scale esophageal ESD may impair the quality of life of patients, stricture prevention after ESD has been of clinical interest. The detailed mechanism underlying this phenomenon has not yet been defined, but massive inflammation and fibrosis after ESD are thought to cause esophageal strictures by reducing elasticity and compliance. Porcine studies have reported that it is possible to reduce esophageal strictures using various sources of mesenchymal stem cells (MSCs).^7^, ^8^ Since MSCs do not directly differentiate into epithelial cells, they are expected to reduce esophageal strictures through their paracrine effects, such as the secretion of angiogenic, anti‐apoptotic, antioxidant, and immunomodulatory factors.^37^, ^38^ This hypothesis can be supported by studies demonstrating similar or even better tissue regeneration effects via infusion or oral ingestion of MSC‐derived conditioned medium compared to MSC transplantation.^8^, ^39^, ^40^ However, a potential limitation of these previous studies was that the paracrine effects of MSCs did not last long with a single injection of stem cells and that conditioned media required repeated oral intake. Therefore, we predicted that using ADSC‐hydrogel mixtures could overcome these shortcomings. Moreover, it would be better if the hydrogels could exist as a liquid before injection and then change to a gel form for a longer period at the initial injection site. Furthermore, in our in vitro experiment, the released amount of paracrine factors from the ADSCs encapsulated in the EISCH hydrogel crosslinked using 6 U/mL of HRP were slightly higher than that from cells encapsulated in the EISCH hydrogel crosslinked using 0.6 U/mL of HRP. We assumed that the mechanically stiffer environment provided by the hydrogel with 6 U/mL of HRP might facilitate the encapsulated cells to release larger amount of paracrine factors than the relatively softer environment provided by the hydrogel with 0.6 U/mL of HRP.^41^, ^42^ In this respect, the ADSC encapsulated in the in vivo‐crosslinked EISCH hydrogel which has similar or slightly higher mechanical property compared to the EISCH hydrogel formed by in vivo‐mimetic crosslinking might be expected to exhibit better paracrine effects.

MSCs can be isolated from various sources such as bone marrow, amniotic fluid, placenta, adult muscle, and corneal stroma as well as adipose tissue. Actually, several studies previously demonstrated that adipose‐derived MSCs (ADSCs) show higher levels of paracrine effects than bone marrow‐derived MSCs for the treatment of ischemic diseases.^43^, ^44^ Thus, we hypothesized that the paracrine secretion of the ADSCs delivered by EISCH contributes to alleviation of esophageal stricture after ESD. Nonetheless, further studies would be required to compare paracrine effects of ADSCs with those of different sources of MSCs.

Finally, a porcine experiment was performed to determine whether it was effective in an environment similar to the clinical setting. The stricture rates were significantly lower in the ADSC and ADSC‐EISCH groups than in the control group. In addition, a significantly milder stricture was observed in the ADSC‐EISCH group than in the ADSC group. To investigate the underlying mechanisms by which stenosis is reduced, immunohistochemical/fluorescence staining with various markers, such as VEGF, Ki‐67, MPO, and α‐SMA, was performed, and the results were compared. VEGF is a growth factor that promotes capillary regeneration in ECs.^45^ Therefore, blood flow around the injected area could be restored, facilitating oxygen support and nutrient delivery. The presence of α‐SMA‐positive myofibroblasts are associated with scar contraction. In healing tissues, fibroblasts are activated to become myofibroblasts and participate in the reparative response by secreting large amounts of extracellular matrix proteins, which may be responsible for wound healing.^46^, ^47^ In the present study, the ADSC and ADSC‐EISCH groups showed decreased α‐SMA expression compared with the controls, with the ADSC‐EISCH group showing much lower α‐SMA expression than the ADSC group. In addition, fewer Ki‐67‐positive cells were detected in the ADSC and ADSC‐EISCH groups than in the control group. Ki‐67 is a proliferation marker used to measure the growth fraction of cells. The significance of Ki‐67 expression is still controversial, but decreased Ki‐67 expression has been reported in a pulmonary fibrosis mouse model after antifibrotic treatment, which is consistent with our results.^48^ Interestingly, the Ki‐67 expression level in the ADSC group at Day 21 seemed to be slightly increased compared to that at Day 14, while the level in the ADSC‐EISCH group stayed about the same. The optimal time of cell retention at the resection site to obtain the best therapeutic effect is still unclear. Given that many patients develop severe stenosis within 2–3 weeks after extensive esophageal ESD, cell retention during at least 2–3 weeks seems to be required. The tendencies of Ki‐67 expression levels over time in the ADSC and ADSC‐EISCH groups might suggest the required period of therapeutic stem cells and emphasize the importance of stable hydrogel scaffold to deliver the cells. Taken together, these results imply that ADSCs could be effective in reducing esophageal stricture, and this effect can be augmented using an ADSC‐EISCH mixture.

Currently, corticosteroids are considered the standard treatment of esophageal stricture after ESD in various ways (either oral or injection, or both) despite potential adverse events such as delayed esophageal perforation, mediastinal abscess, and steroid‐induced side effects.

Treatment of esophageal stricture using polyglycolic acid (PGA) sheets has been reported,^49^, ^50^, ^51^ but the mechanism preventing the stricture is still elucidated yet. It has been speculated that the PGA sheet protects the wound from external stimuli, leading to less stricture. Compared with these conventional methods, stem cell transplantation using injectable hydrogel seems to be more effective for prevention of esophageal stricture since MSCs secrete a wide spectrum of paracrine factors for not only inhibiting inflammation and abnormal tissue growth but also enhancing tissue regeneration and angiogenesis.^52^, ^53^, ^54^ Nonetheless, regenerative approaches using ADSC or ADSC‐hydrogel mixture are still experimental and have not yet been approved for human use because long‐term efficacy and safety need to be carefully investigated. Direct comparison study between current treatments such as corticosteroid or PGA sheets and our method is required to evaluate the treatment effect in the future. Further biological analysis like time‐course spatial transcriptomics would be required to determine the molecular mechanism underlying the effect of stem cell‐hydrogel hybrid system for prevention of esophageal strictures. Additionally, hydrogel‐mediated delivery of engineered stem cells to overexpress angiogenic and/or anti‐inflammatory factors would enable prolonged cell retention and further enhanced paracrine effects for more effectively preventing esophageal strictures.

This study had several limitations. First, the number of animals used in this study was small. However, a similar number of animals was used in most studies using porcine models. Considering the principles of the 3Rs (replacement, reduction, and refinement) in animal research, the sample size in this study could still be considered appropriate. Second, since this study used small young pigs (20–25 kg) that were still growing, it is unclear whether the results can be inferred for human adults. However, because the stomach and esophagus of pigs of this size and age are similar to those of humans, using pigs of similar sizes is preferred in most studies using pig models. Finally, unlike humans, it was difficult to strictly control the diet of pigs after ESD. As water was allowed to be consumed freely, it cannot be excluded that a part of the injected ADSCs‐hydrogel mixture was washed out, underestimating its therapeutic effect.

## 5 CONCLUSION

In conclusion, our study demonstrated that the acsidian‐inspired hyaluronic acid hydrogel system could be applied effectively to the endoscopic injection of therapeutic stem cells owing to its improved injectability attributed to the in situ self‐crosslink‐ability and superior biocompatibility. Thus, the mixtures containing the endoscopically injectable hydrogel and ADSCs significantly reduced post‐ESD esophageal stricture by augmenting the paracrine effects of ADSCs. Although further investigation and comparative studies are required to confirm the clinical application of this system, our injectable hydrogel‐based therapeutic cell delivery system may suggest an innovative platform for cell therapies using familiar and established medical equipment in more clinically relevant situations.

## AUTHOR CONTRIBUTIONS

**Hyunsoo Chung:** Conceptualization (equal); data curation (equal); formal analysis (equal); funding acquisition (equal); investigation (equal); methodology (equal); validation (equal); visualization (equal); writing – original draft (equal); writing – review and editing (equal). **Soohwan An:** Conceptualization (equal); data curation (equal); formal analysis (equal); investigation (equal); methodology (equal); software (equal); validation (equal); visualization (equal); writing – original draft (equal); writing – review and editing (equal). **Seung Yeop Han:** Investigation (supporting); methodology (supporting). **Jihoon Jeon:** Investigation (supporting); methodology (supporting). **Seung‐Woo Cho:** Conceptualization (equal); funding acquisition (equal); project administration (equal); supervision (equal); writing – review and editing (equal). **Yong Chan Lee:** Conceptualization (equal); project administration (equal); supervision (equal); writing – review and editing (equal).

## CONFLICT OF INTEREST STATEMENT

All authors declare that there are no conflicts of interest related to this work.

## Supporting information


**Figure S1.** Chemical analysis for confirming synthesis of EISCH. (a) ^1^H‐NMR spectrum and (b) UV‐vis spectrum of EISCH.
**Figure S2.** Rheological analysis of EISCH hydrogel. Storage and loss modulus measured in a frequency sweep mode at a range of 0.1–1 Hz.
**Figure S3.** Cell viability test after injection of cell‐loaded EISCH. (a) Fluorescent images from the live/dead assay and (b) the viability of ADSCs in the EISCH hydrogels after passing the cell‐loaded EISCH pre‐gel solution through different gauge of needles (*n* = 10, independent samples through separate cell experiments). Control group was prepared by only pipetting without passing through the needles.
**Figure S4.** Histological analysis of hADSCs‐loaded EISCH‐injected esophageal tissues in a pig. (a) H&E and (b) MT‐stained images of hADSCs‐loaded EISCH‐injected esophageal tissues in a pig after 7 and 21 days of injection. (c) Fluorescence images of the dotted area in the MT‐stained images (hADSC‐loaded EISCH‐injected region) (scale bar = 100 μm).
**Figure S5.** Time‐dependent changes of Q‐dots (QD) signals. The QD655‐labeled ADSC observed in esophageal tissues in ADSC group and ADSC‐EISCH group after 7, 14, and 21 days of injection.

## Data Availability

The data that support the findings of this study are available from the corresponding authors upon reasonable request.
