# Supplementary material for: Endoscopically injectable and self‐crosslinkable hydrogel‐mediated stem cell transplantation for alleviating esophageal stricture after endoscopic submucosal dissection
Source: Bioeng Transl Med. 2023 Apr 18;8(3):e10521. doi: 10.1002/btm2.10521 (PMC10189443; doi:10.1002/btm2.10521)
Supplement: Supplementary file 1 — Figure S1. Chemical analysis for confirming synthesis of EISCH. (a) 1H‐NMR spectrum and (b) UV‐vis spectrum of EISCH. Figure S2. Rheological analysis of EISCH hydrogel. Storage and loss modulus measured in a frequency sweep mode at a range of 0.1–1 Hz. Figure S3. Cell viability test after injection of cell‐loaded EISCH. (a) Fluorescent images from the live/dead assay and (b) the viability of ADSCs in the EISCH hydrogels after passing the cell‐loaded EISCH pre‐gel solution through different gauge of needles (n = 10, independent samples through separate cell experiments). Control group was prepared by only pipetting without passing through the needles. Figure S4. Histological analysis of hADSCs‐loaded EISCH‐injected esophageal tissues in a pig. (a) H&E and (b) MT‐stained images of hADSCs‐loaded EISCH‐injected esophageal tissues in a pig after 7 and 21 days of injection. (c) Fluorescence images of the dotted area in the MT‐stained images (hADSC‐loaded EISCH‐injected region) (scale bar = 100 μm). Figure S5. Time‐dependent changes of Q‐dots (QD) signals. The QD655‐labeled ADSC observed in esophageal tissues in ADSC group and ADSC‐EISCH group after 7, 14, and 21 days of injection. [file BTM2-8-e10521-s001.doc]

Supporting Information

**Endoscopically injectable and self-crosslinkable hydrogel-mediated stem cell transplantation for alleviating esophageal stricture after endoscopic submucosal dissection**

*Hyunsoo Chung^1,2,3†^, Soohwan An^4†^, Seung Yeop Han^4^, Jihoon Jeon^4^, Seung-Woo Cho^4,5,6*^, Yong Chan Lee^3,7*^*

^1^Department of Internal Medicine and Liver Research Institute, Seoul National University College of Medicine, Seoul, Republic of Korea

^2^Department of Medical Device Development, Seoul National University College of Medicine, Seoul, Republic of Korea

^3^Yonsei University Graduate School of Medicine, Seoul, Republic of Korea

^4^Department of Biotechnology, Yonsei University, Seoul, Republic of Korea

^5^Center for Nanomedicine, Institute for Basic Science (IBS), Seoul, Republic of Korea

^6^Graduate Program of Nano Biomedical Engineering (NanoBME), Advanced Science Institute, Yonsei University, Seoul, Republic of Korea

^7^Department of Internal Medicine, Yonsei University College of Medicine, Seoul, Republic of Korea

**^†^**These authors contributed equally to this work

^*^Co-corresponding authors

**Correspondence**

Seung-Woo Cho, PhD

Department of Biotechnology

Yonsei University

Seoul 03722, Republic of Korea

E-mail: [seungwoocho@yonsei.ac.kr](mailto:seungwoocho@yonsei.ac.kr)

Yong Chan Lee, MD, PhD

Department of Internal Medicine

Severance Hospital, Yonsei University College of Medicine

Seoul 03722, Republic of Korea

E-mail: [leeyc@yuhs.ac](mailto:leeyc@yuhs.ac)


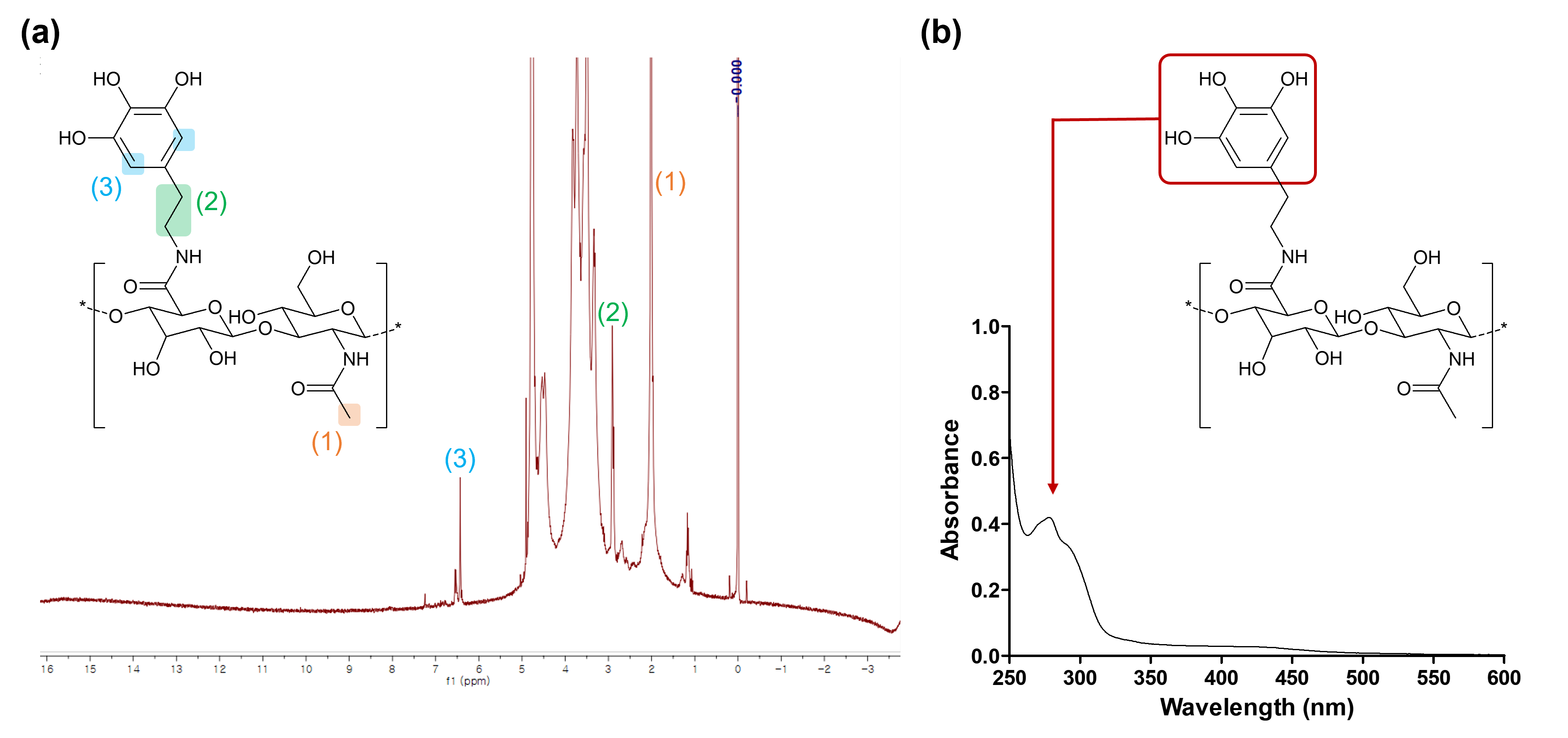


**Figure S1.** **Chemical analysis for confirming synthesis of EISCH.** (a) ^1^H-NMR spectrum and (b) UV-vis spectrum of EISCH.


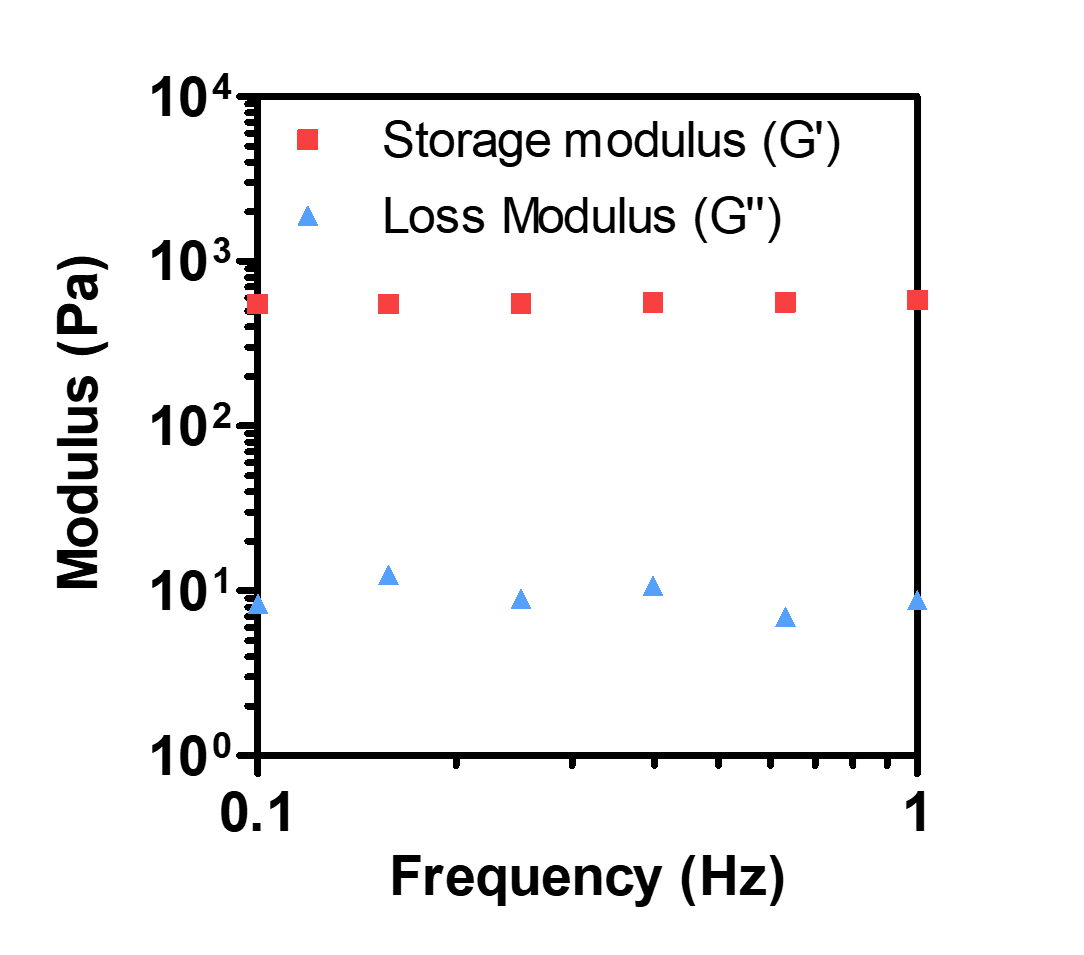


**Figure S2.** **Rheological analysis of EISCH hydrogel.** Storage and loss modulus measured in a frequency sweep mode at a range of 0.1 – 1 Hz.


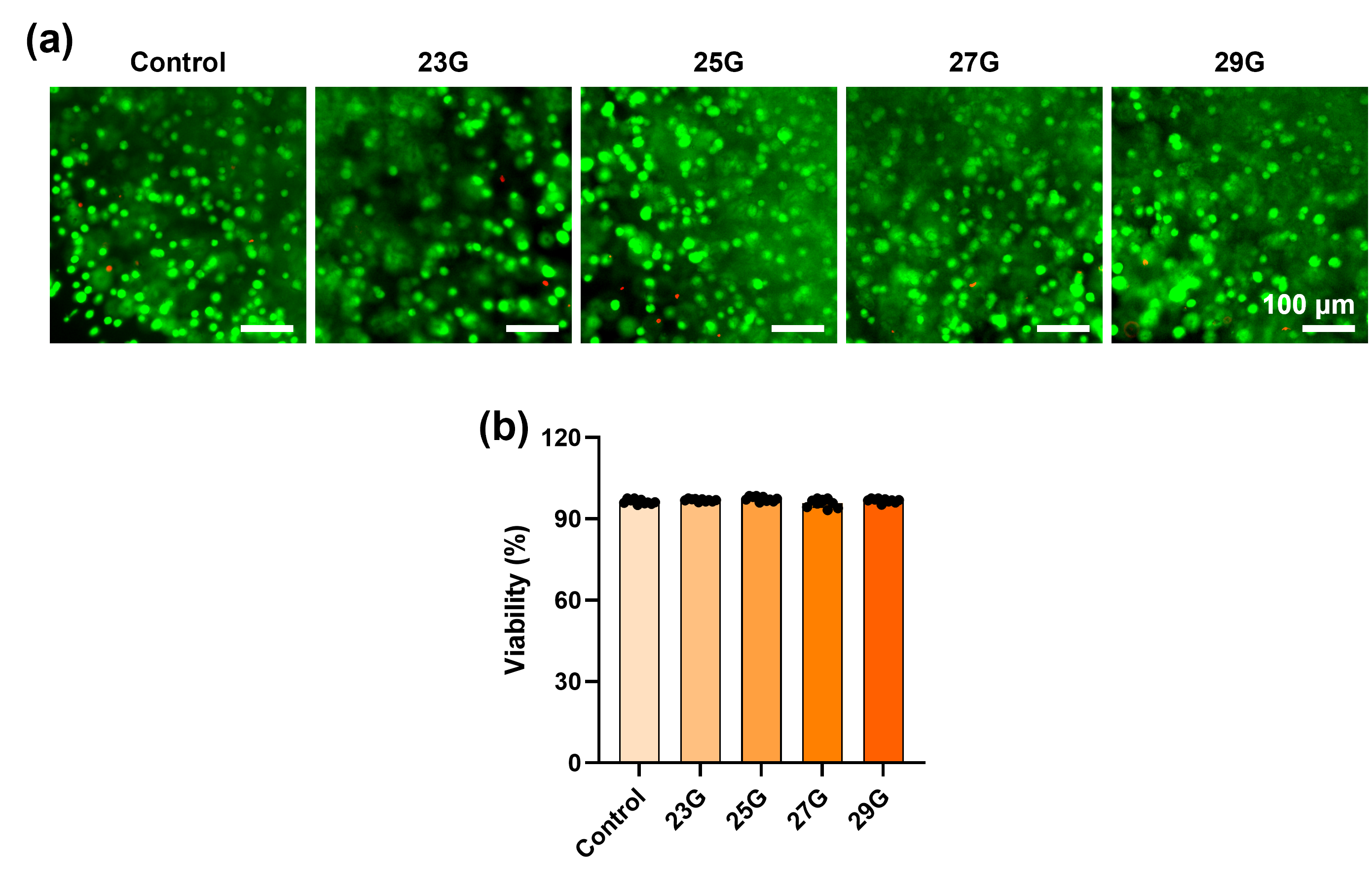


**Figure S3. Cell viability test after injection of cell-loaded EISCH.** (a) Fluorescent images from the live/dead assay and (b) the viability of ADSCs in the EISCH hydrogels after passing the cell-loaded EISCH pre-gel solution through different gauge of needles (*n* = 10, independent samples through separate cell experiments). Control group was prepared by only pipetting without passing through the needles.


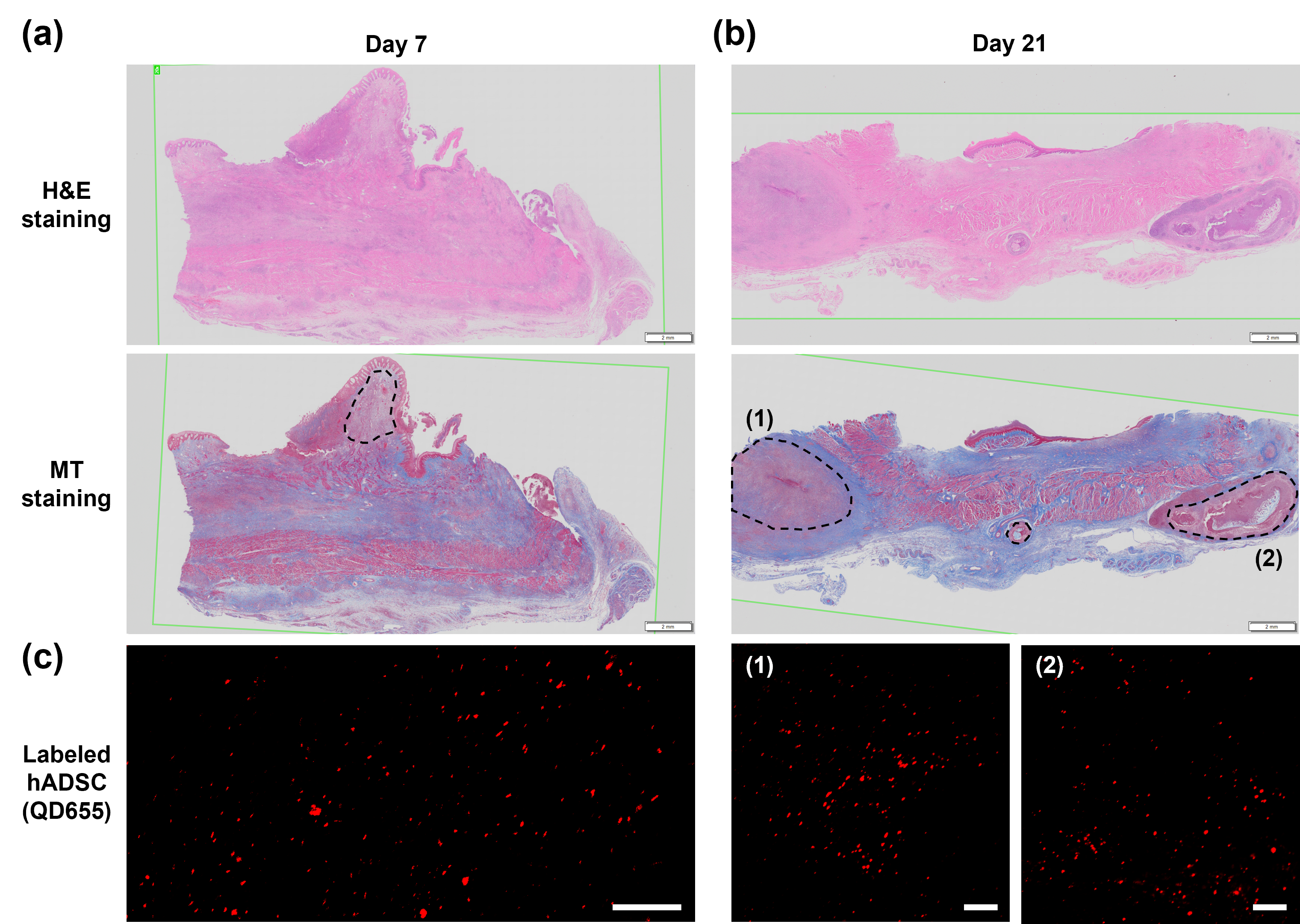


**Figure S4.** **Histological analysis of hADSCs-loaded EISCH-injected esophageal tissues in a pig.** (a) H&E and (b) MT-stained images of hADSCs-loaded EISCH-injected esophageal tissues in a pig after 7 and 21 days of injection. (c) Fluorescence images of the dotted area in the MT-stained images (hADSC-loaded EISCH-injected region) (scale bar = 100 μm).


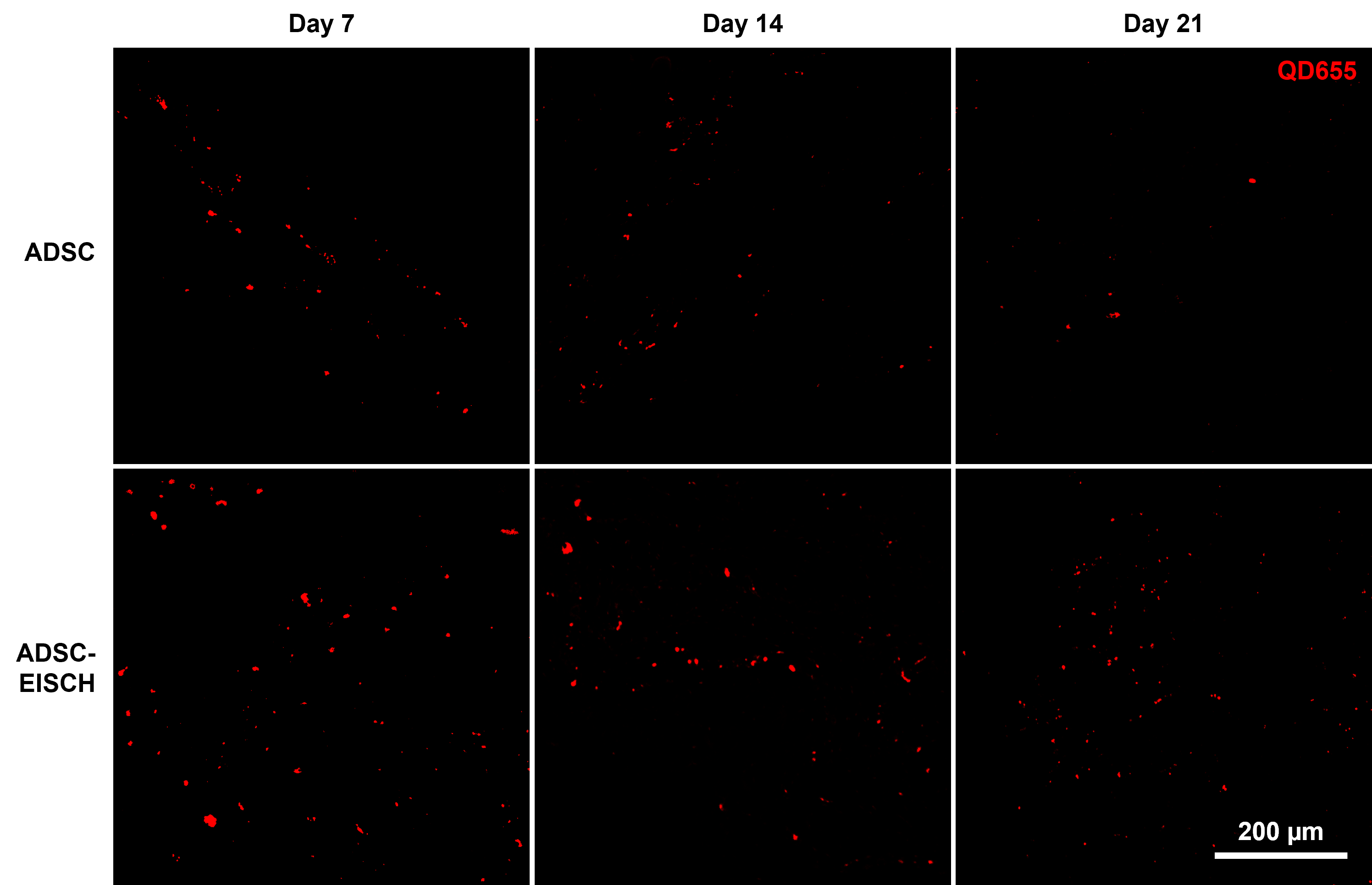


**Figure S5.** **Time-dependent changes of Q-dots (QD) signals.** The QD655-labelled ADSC observed in esophageal tissues in ADSC group and ADSC-EISCH group after 7, 14 and 21 days of injection.
